# *Lyar* contributes to cell cycle progression and multi-lineage differentiation in mouse embryonic stem cells

**DOI:** 10.3389/fgene.2026.1786528

**Published:** 2026-03-20

**Authors:** Yuanqing Pan, Yuqi Su, Li Xing, Mingze Yao

**Affiliations:** 1 Biomedical and Health Laboratory in Shanxi Province, Institutes of Biomedical Sciences, Shanxi University, Taiyuan, China; 2 Key Laboratory of Chemical Biology and Molecular Engineering of Ministry of Education, Institutes of Biomedical Sciences, Shanxi University, Taiyuan, China

**Keywords:** differentiation, embryoid body, embryonic stem cells, knockout, *Lyar*, proliferation

## Abstract

Ly-1 antibody reactive clone gene (*Lyar*) is involved in the regulation of embryonic stem cell (ESC) self-renewal. To explore the specific role of *Lyar* in cell cycle progression and embryonic differentiation, we generated *Lyar* knockout (KO) mouse ESC (mESC) lines using CRISPR/Cas9, and investigated the effects of *Lyar* deficiency on mESC proliferation, cell cycle, apoptosis and multi-lineage differentiation. We found that *Lyar* deficiency reduces proliferation, increases apoptosis, and elevates p53 and p21 protein expression. The impaired mESC proliferation is associated with the increased apoptosis and cell cycle progression defect, which is driven by p53-p21 pathway activation. In embryoid body (EB) formation assay, loss of *Lyar* led to significant downregulation of most germ layer-specific markers in KO mESC clones, including mesoderm (*Gsc*, *T*), endoderm (*Gata4*, *Sox17*) and ectoderm marker *Pax6*. These findings confirm that *Lyar* is required for cell cycle progression, proliferation, and lineage-specific marker expression during early differentiation, demonstrating that *Lyar* may serve as a critical regulatory factor in stem cell biology.

## 1 Introduction

Mouse embryonic stem cells (mESCs), derived from the inner cell mass (ICM) of pre-implantation embryos (Evans and Kaufman, 1981; Martin, 1981), possess the capacity for self-renewal and differentiation into all cell types of an organism (pluripotency). A classic study by Niwa and colleagues demonstrated that the precise expression level of *Oct4* acts as a molecular switch dictating mESC fate by governing self-renewal, differentiation, or dedifferentiation, highlighting the critical role of quantitative control of transcription factors in maintaining pluripotency (Niwa et al., 2000).

Beyond core transcription factors, nucleolar proteins are being increasingly recognized as key regulators of stem cell fate. For instance, Nucleolin maintains mESC self-renewal by suppressing the p53-dependent pathway, thereby promoting cell cycle progression and proliferation (Yang et al., 2011). Similarly, depletion or overexpression of nucleostemin impairs proliferation in central nervous system (CNS) stem cells or transformed cells (Tsai and McKay, 2002).

*Lyar* (Ly-1 antibody reactive clone) encodes another nucleolar protein that contains 388 amino acids and harbors a LYAR-type C2HC zinc finger motif and three nuclear localization signals. *Lyar* was first identified in a mouse T-cell leukemia line and is abundantly expressed in immature spermatocytes within the testis (Su et al., 1993). Previous studies have shown that knockdown of *Lyar* impairs cell growth, enhances apoptosis, and disrupts the balance between self-renewal and differentiation, suggesting that *Lyar* may play a role in maintaining ESC identity (Li et al., 2009). However, the phenotypic consequences of complete *Lyar* loss-of-function in mESCs were not characterized. To address this gap, we generated *Lyar* knockout (KO) mESCs and systematically examined their proliferation, cell cycle progression, and differentiation potential. Our findings offer critical insights into *Lyar*’s potential physiological role in mESC biology.

## 2 Materials and methods

### 2.1 Culture of mESCs

Mouse T4 ESCs (Zhang et al., 2025) were a kind gift of Dr. Shuai Ling (Nankai University). Mouse ESCs were cultured on 0.1% gelatin-coated plates and in ES medium: DMEM medium supplemented with 20% fetal bovine serum (FBS), leukemia inhibitory factor (LIF, Sino Biological lnc), 0.1 mM 2-mercaptoethanol, 1 mM non-essential amino acids (Gibco), 1 × GlutaMAX (Gibco), 1 mM sodium pyruvate (Gibco), 100 U/mL Penicillin, 100 μg/mL Streptomycin (Hyclone), and 2i inhibitors (3 μM CHIR99021 and 1 μM PD0325901). ESCs were passaged with 0.25% Trypsin/EDTA treatment and were confirmed to be free from *mycoplasma* contamination.

### 2.2 Generation of *Lyar* knockout mESCs

The donor plasmid pMD-18T (containing the PGK-Puro-P2A-mCherry cassette flanked by left and right homology arms) and two pX330 plasmids (expressing sgRNAs targeting the sequences 5′- gaa​acc​caa​cgt​cag​ccc​ca-3′ and 5′-cag​cga​ctc​cgt​tct​aga​gc-3′ upstream of Exon 3 and downstream of Exon 4 of *Lyar*, respectively) were constructed. These sgRNAs were selected from the Mouse CRISPR Knockout Pooled Library (GeCKO v2) and designed to disrupt exons encoding critical functional domains, ensuring frameshift mutation and complete loss of function of *Lyar*. The donor plasmid and the two sgRNA-expressing plasmids were mixed at a ratio of 2:1:1 (total 2.5 μg) and electroporated into 2.5 × 10^5^ mESCs using the P3 Primary Cell 4D-Nucleofector™ X Kit L (V4XP-3012, Lonza) according to the manufacturer’s instructions. The transfected cells were subsequently selected in medium containing 1 μg/mL puromycin (Gibco) for 7 days. After two passages, individual clones were picked when the cell density reached approximately 60% and plated onto gelatin-coated 24-well plates (one clone per well). Genomic DNA was extracted using the Tiangen Genomic DNA Kit (Tiangen, Beijing, China) and PCR was performed using Genstar LongTaq PCR StarMix on a Veriti™ 96-Well Fast Thermal Cycler (Thermo Fisher) under the following conditions: initial denaturation at 95 °C for 3 min, followed by 35 cycles of 95 °C for 15 s, 60 °C for 15 s, and 72 °C for 30 s. Genomic PCR was used to confirm the correct integration of the PGK-Puro-P2A-mCherry cassette and the deletion of genomic sequences between the third and fourth exons of *Lyar*. The forward primer F (5′-TGT​TTG​TCA​CTA​TGC​ATA​GAT​G-3′) and reverse primer R (5′-CTG​GAA​ATG​CCA​TCT​TGA​TC-3′) were used in PCR verification.

### 2.3 Karyotype determination

G-banding analysis was performed to evaluate chromosomal integrity. mESCs were seeded on Matrigel-coated 6-well plates and grown to 70% confluency. Colchicine (0.2 μg/mL) was added for 2 h to arrest cells in metaphase. Cells were then detached with 0.5 mM EDTA for 5 min, collected, and centrifuged at 1,000 rpm for 5 min. The cell pellets were subjected to hypotonic treatment with 0.075 M KCl (37 °C, 20 min) and fixed in 3:1 methanol/acetic acid (37 °C, 10 min). After centrifugation, the supernatant was discarded, and the cell pellet was resuspended in ice-cold fixative. The cell suspension was dropped onto chilled glass slides, air-dried, and incubated at 80 °C for 2 h. Chromosomes were then treated with trypsin and stained with Giemsa. Images of metaphase chromosomes were captured using an Olympus BX51 Microscope and analyzed with the Ikaros Karyotyping System (MetaSystems).

### 2.4 Quantitative real-time PCR (qPCR)

RNA was extracted using the EZ-press RNA Purification Kit (EZBioscience). The cDNA was synthesized using a reverse transcriptase kit (Vazyme). Real-time quantitative polymerase chain reaction (qPCR) was performed using a SYBR Premix Ex Taq Kit (Takara, catalog no. CN830S) and a CFX96 Touch Real-Time PCR System (Bio- Rad).

Primers used in qPCR are for following genes: *Nanog* (Forward: 5′-CTCAAGTCCTGAGGCTGACA-3′, Reverse: 5′-TGA​AAC​CTG​TCC​TTG​AGT​GC-3′), *Oct4* (Forward: 5′-TAGGTGAGC CGTCTTTCCAC-3′, Reverse: 5′- GCT​TAG​CCA​GGT​TCG​AGG​AT-3′), *Gata4* (Forward: 5′-CCCTAC​CCA​GCC​TAC​ATG​G-3′, Reverse: 5′-ACA​TAT​CGA​GAT​TGG​GGT​GTC​T-3′), *Sox17* (Forward: 5′-CGA​GCC​AAA​GCG​GAG​TCT​C-3′, Reverse: 5′-TGC​CAA​GGT​CAA​CGC​CTT​C-3′), *Gsc* (Forward: 5′-ACC​ATC​TTC​ACC​GAT​GAG​CAG​C-3′, Reverse: 5′-CTT​GGC​TCG​GCG​GTT​CTT​AAAC-3′), *T* (Forward: 5′-GCT​TCA​AGG​AGC​TAA​CTA​ACG​AG-3′, Reverse: 5′-CCAGCAAGAAAGAGTACATGGC-3′), *Pax6* (Forward: 5′-GCA​GAT​GCA​AAA​GTC​CAG​GTG-3′, Reverse: 5′-CAG​GTT​GCG​AAG​AAC​TCT​GTT​T-3′), *Nestin* (Forward: 5′-CCC​TGA​AGT​CGA​GGA​GCT​G-3′, Reverse: 5′-CTG​CTG​CAC​CTC​TAA​GCG​A-3′).

### 2.5 Immunofluorescence staining

Cells were fixed with 4% paraformaldehyde (Beyotime, China) for 20 min, permeabilized with 0.3% Triton X-100 (Solarbio, China, catalog no. T8200) for 10 min, blocked with 5% bovine serum albumin, and incubated with primary antibodies overnight at 4 °C. Then, cells were incubated with secondary antibodies labeled with Alexa Fluor™ 488-A-11034 (1:1000; Thermo Fisher Scientific), and 4′,6-diamidino-2-phenylindole (Beyotime, catalog no. C1005). Confocal images were acquired using a ZEISS LSM 710 confocal microscope (ZEISS, China). The primary antibodies used include anti-OCT4 (Proteintech, catalog no. 11263-1-AP), anti-NANOG (Proteintech, catalog no. 14295-1-AP) and LYAR Rabbit Polyclonal antibody (Proteintech, 24433-1-AP).

### 2.6 Western blotting analysis

Total proteins were extracted in RIPA lysis buffer, separated in 12% Sodium dodecyl sulfate polyacrylamide gel electrophoresis (SDS-PAGE), transferred onto nitrocellulose membrane, incubated with primary antibody overnight at 4 °C, and then washed with Tris-buffered saline with 0.1% Tween® 20 detergent (TBST) three times (each wash for 5 min). The membranes were then incubated with horseradish peroxidase (HRP)-conjugated secondary antibody at room temperature for 1 h, followed by TBST washes. Protein bands were visualized using an enhanced chemiluminescence (ECL) detection kit (Epizyme) and images were visualized using Bio-Rad chemiluminescence imaging system.

The primary antibodies used include LYAR Rabbit Polyclonal antibody (Proteintech, 24433-1-AP), Cyclin D1 Rabbit Polyclonal antibody (Zhengneng, 382442), Anti-CDKN2A/p16INK4a Rabbit Monoclonal antibody (Epizyme, R010616), p21 Rabbit Polyclonal antibody (wanlei Bio, WL0362), p53 Rabbit Polyclonal antibody (Wanlei Bio, WL01919), β-actin Mouse Monoclonal Antibody (Epizyme, LF201) and GAPDH Mouse Monoclonal Antibody (Epizyme, LF205S). Horseradish peroxidase (HRP)-conjugated Goat Anti-Rabbit IgG (Proteintech, SA00001-2) and HRP-conjugated Goat Anti-Mouse IgG (Proteintech, SA00001-1) were used as secondary antibodies.

Uncropped immunoblots are provided in the Supplementary Material. Band intensities were quantified using ImageJ software.

### 2.7 Embryoid body (EB) formation

Hanging drops (1,000 cells per drop) were used to culture cells to form embryoid body (Suchý et al., 2021) in a differentiation medium. The medium was prepared without LIF and 2i inhibitors. After 4 days, the EBs were transferred into 100 mm Petri dishes in a differentiation medium for 2 days. Total RNA was then extracted, and the expression of marker genes for three germs were subsequently detected by qPCR.

### 2.8 CCK-8 proliferation assay

Cell proliferation was assessed by performing CCK8 experiment with a Cell Counting Kit-8 (APExBIO, K1018) according to the instruction of manufactory. 2000 cells were cultured on a 96-well culture plate. At days 1, 2, 3, 4, and 5, 10 μL CCK8 was added into 100 μL culture medium at 37 °C for 2 h. The absorbance was measured at 450 nm using a multifunctional microporous detector (BioTek, United States).

### 2.9 EdU assay

The cells were seeded into 12-well plates at a density of 3 × 10^4^ cells per well and cultured at 37 °C with 5% CO_2_. After 3.5 days, cell cycle stage was determined using BeyoClick™ EdU Cell Proliferation Kit with Alexa Fluor 488 (Beyotime, C0071S) according to the manufacturer’s instructions. Briefly, cells were incubated with EdU (10 μM final concentration) for 30 min, digested using 0.25% trypsin-EDTA, fixed in 4% PFA followed by permeabilization in PBS/0.3% Triton X-100 at room temperature for 15 min. After washing three times using washing buffer (PBS/3% BSA), click reaction solution was added into the tube and incubated in the dark for 30 min at room temperature. Next, the cell suspension was washed in washing solution and stained with DAPI for 10 min at room temperature. Samples were then analyzed using LSRFortessa (BD Biosciences).

### 2.10 Apoptosis detection

The cells were seeded into 12-well plates at a density of 4.5 × 10^4^ cells per well. After 48 h, cell apoptosis was detected using an APC Annexin V/7-AAD Apoptosis Detection Kit (Elabscience, E-CK-A218). After trypsinization and washing, cells were resuspended in binding buffer and stained with APC Annexin V and 7-AAD in the dark for 15 min at room temperature. Stained cells were analyzed by flow cytometer (BECKMAN COULTER) to determine the proportion of apoptotic cells. Early apoptotic cells were defined as APC Annexin V-positive and 7-AAD-negative, while late apoptotic or necrotic cells were APC Annexin V-positive and 7-AAD-positive. Each experiment was repeated three times. To avoid fluorescence interference from mCherry, appropriate single-stain controls were prepared for compensation, and fluorescence signals were collected using optimized filter settings to ensure accurate quantification.

### 2.11 Statistical analysis

The data were presented as mean ± SEM. The statistical analyses were performed using GraphPad software and the experiments were performed in technical triplicates (n = 3). A two-tailed *t*-test was performed to compare the statistical significance.

## 3 Results

### 3.1 Establishing mESCs with functional loss of *Lyar*

**FIGURE 1:**
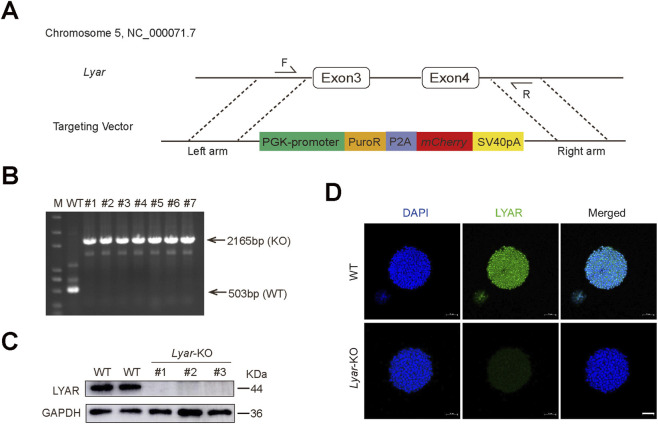
Establishing mESCs with functional loss of *Lyar*. **(A)** Schematic of *Lyar* gene targeting design. Depicted are the partial *Lyar* gene (Chr 5: NC_000071.7, Exon 3–4) and its targeting vector, which carries left/right homologous arms (flanking Exon 3-4, matching PCR primer F/R sites), plus a PGK-driven Puro-P2A-mCherry cassette for *Lyar* locus modification. Primers F (Exon 3 upstream) and R (Exon 4 downstream) amplify the Exon 3-4 fragment. **(B)** PCR genotyping of *Lyar* locus in WT and clones 1–7: The 2165bp band indicates knockout (KO) allele, and 503bp band indicates WT allele. M, DNA ladder marker. **(C)** Western blot analysis showing expression levels of LYAR in WT and *Lyar*-KO mESCs. Full uncropped blots are provided in the Supplementary Material. **(D)** Immunostaining of LYAR for WT and *Lyar*-KO mESCs. Scale bar, 50 μm.

Although previous knockdown studies suggested a role for *Lyar* in mESC biology, the effects of complete *Lyar* ablation remained unclear. To fully elucidate *Lyar*’s function, we generated *Lyar*-KO mESCs using CRISPR/Cas9-mediated genome editing. A targeting vector (donor plasmid) was constructed, containing left and right homology arms flanking a PGK-Puro-P2A-mCherry cassette. This donor vector was co-electroporated into wild-type (WT) mESCs along with two sgRNA-PX330 plasmids. These sgRNAs were designed to target the intronic regions upstream of the third exon and downstream of the fourth exon of *Lyar*, guiding Cas9 to generate two double-strand breaks (DSBs). Through homologous recombination, the genomic region spanning exons 3 and 4 was replaced by the PGK-Puro-P2A-mCherry cassette (Figure 1A). Transfected cells were selected with puromycin, and individual clones were picked for following experiments.

Next, the *Lyar* knockout efficiency was validated in mESCs at both genomic and protein levels. For genomic verification, PCR genotyping was performed on WT and knockout clones 1–7 (Figure 1B): the 503 bp band (wild-type allele) was detected in WT, while the 2165 bp band (knockout allele with exogenous cassette integration) was specifically present in clones 1–7, confirming successful deletion of the target *Lyar* fragment and precise insertion of the exogenous cassette in these clones. Western blot analysis (Figure 1C) showed that LYAR protein was abundantly expressed in WT cells, but completely absent in *Lyar*-KO clones. This loss of LYAR expression was further corroborated by immunofluorescence assays (Figure 1D) where distinct LYAR-specific green fluorescence was observed in WT cells, but not in *Lyar*-KO cells. These results collectively demonstrate that the *Lyar* gene was successfully replaced by the exogenous cassette in mESC clones, leading to complete loss of LYAR protein expression. For subsequent functional assays, clone 1 served as the primary material for CCK-8 analysis, and both clone 1 and clone 2 were tested for EdU incorporation, apoptosis, and EB differentiation assays to exclude clonal variation.

### 3.2 Effects of *Lyar* knockout on the genomic stability

**FIGURE 2:**
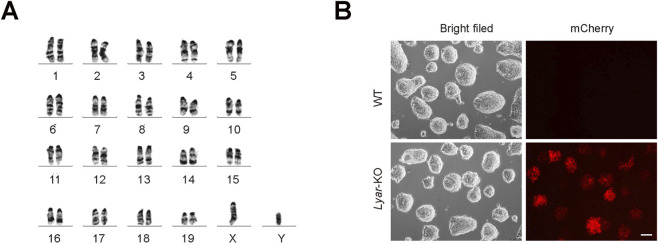
Effects of *Lyar* knockout on the genomic stability. **(A)** Karyotype analysis of *Lyar*-KO mESCs after 12 passages. The *Lyar* KO clone exhibits a normal diploid karyotype (40 chromosomes, XY), consistent with WT mESC genetic background. **(B)** Morphological and fluorescence analysis of *Lyar*-KO mESCs. Bright-field images show that *Lyar*-KO mESCs maintain typical mESC colony morphology identical to WT cells. Fluorescence images reveal robust mCherry expression in *Lyar*-KO cells (but not in WT cells), confirming successful integration of the PGK-Puro-P2A-mCherry cassette. Scale bar, 100 μm.

To assess the genetic and morphological stability of the established *Lyar* KO mESC line, we performed karyotype analysis. The results showed a normal diploid karyotype (40 chromosomes, XY), consistent with the WT mESC background (Figure 2A), confirming that loss of *Lyar* did not cause detectable chromosomal abnormalities. Morphologically, *Lyar*-KO mESCs retained the typical compact, dome-shaped morphology of undifferentiated mESCs (Figure 2B). Additionally, strong mCherry fluorescence was exclusively observed in the *Lyar* KO line (but not in WT cells), further verifying the successful integration and expression of the PGK-Puro-P2A-mCherry cassette. Taken together, these results demonstrate that the established *Lyar* KO mESC line is genetically stable and maintains normal ESC morphology.

### 3.3 *Lyar* is required for efficient proliferation of mESCs

**FIGURE 3:**
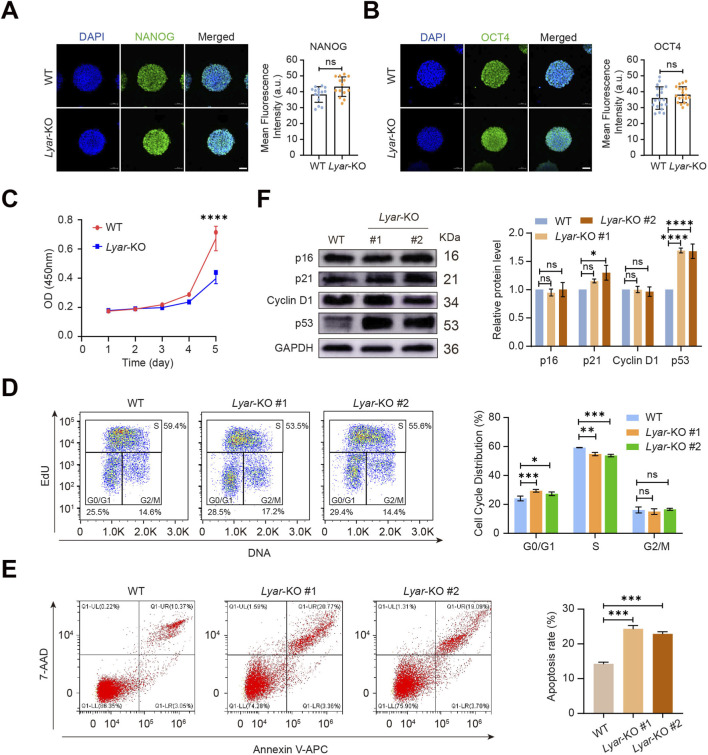
*Lyar* is required for efficient proliferation of mESCs. Immunofluorescence staining of pluripotent marker NANOG **(A)** and OCT4 **(B)** in WT and *Lyar*-KO mESCs (left panels), and quantification of their mean fluorescence intensity (right panels). Nuclei were counterstained with DAPI (blue). Scale bar, 50 μm. Data are presented as mean fluorescence intensity (arbitrary units, a.u.) ± SEM. Each dot represents an individual mESC colony. ns, not significant (Student’s t-test). **(C)** CCK-8 assay showing the proliferation rates of WT and *Lyar-*KO clone 1. **(D)** Cell cycle analysis. Left, representative flow cytometry dot plots showing EdU incorporation in WT and *Lyar-*KO clone 1 and clone 2; Right, quantification of EdU-positive cells. **(E)** Apoptosis analysis by Annexin V-APC/7-AAD staining. Left, representative flow cytometry plots of WT, *Lyar*-KO clone 1, and *Lyar*-KO clone 2. Right, quantification of the apoptotic rate. **(F)** Western blot analysis of key G1/S checkpoint regulators, including p53, p21, Cyclin D1, and p16, in WT, *Lyar*-KO clone 1, and *Lyar*-KO clone 2. Representative blots are shown. Full uncropped blots are provided in the Supplementary Material. Data are presented as mean ± SEM (n = 3) and the significance level was calculated by Student’s t test (two-tailed, equal variance) (*, *p* < 0.05; **, *p* < 0.01; ***, *p* < 0.001; ****, *p* < 0.0001).

To determine the effects of *Lyar* depletion on mESC pluripotency, the expression of core pluripotency markers (Niwa et al., 2000; Ni et al., 1998; Chambers et al., 2003; Mitsui et al., 2003) were evaluated. Immunofluorescence staining revealed that *Lyar*-KO mESCs retained robust expression of NANOG and OCT4, consistent with the typical expression pattern in WT mESCs (Figures 3A,B). These results are consistent with previous reports in *Lyar* knockdown cells, indicating that *Lyar* loss does not impair the expression of core pluripotency factors in mESCs. The effects of *Lyar* loss on cell proliferation and cell cycle progression were tested using CCK-8 and EdU incorporation assays, respectively. CCK-8 proliferation assays showed that *Lyar*-KO clone 1 exhibited a significant reduction in proliferation rate compared to WT controls (Figure 3C). Consistent with the proliferation defect, EdU incorporation assays revealed a significant increase in the G0/G1 population and decrease in the percentage of S-phase cells in both *Lyar*-KO clone 1 and clone 2 compared with WT mESCs (Figure 3D). Given that impaired cell cycle progression is often associated with altered cell survival, we further examined apoptosis in *Lyar*-deficient cells using flow cytometry. Notably, the apoptotic rate was significantly increased in both *Lyar*-KO clone 1 and clone 2 relative to WT cells (Figure 3E), suggesting that the loss of *Lyar* compromises cell survival in mESCs.

To explore the molecular mechanism underlying the cell cycle progression defect, we assessed the expression of key regulatory proteins governing the G1/S checkpoint, including p53, p21, Cyclin D1, and p16. In *Lyar*-KO clones 1 and 2, p53 and its downstream target p21 were markedly elevated compared with WT cells, whereas the expression levels of Cyclin D1 and p16 remained unchanged (Figure 3F). These results demonstrate that *Lyar* deficiency may activate the p53-p21 signaling axis, which in turn inhibits cell cycle progression and contributes to the impaired proliferation and cell cycle defects observed in *Lyar*-deficient mESCs.

### 3.4 Loss of *Lyar* alters the expression of lineage-specific markers during embryoid body differentiation of mESCs

**FIGURE 4:**
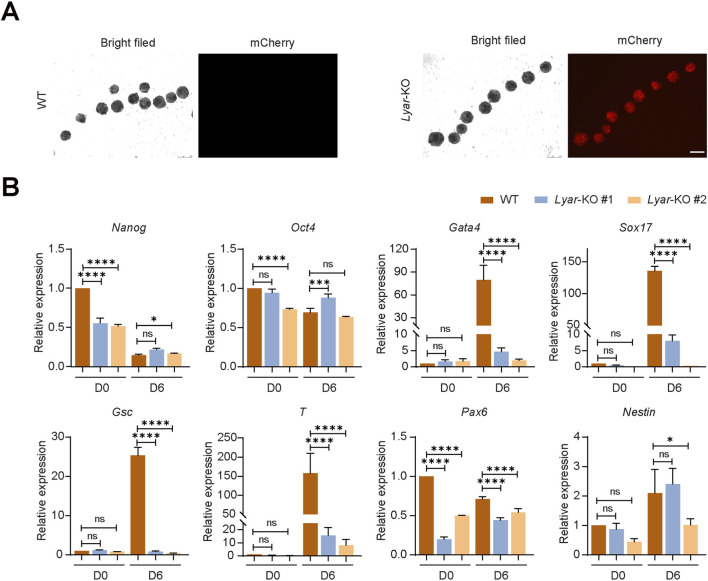
Loss of *Lyar* alters the expression of lineage-specific markers during embryoid body differentiation of mESCs. **(A)** Morphology and mCherry expression of EBs derived from *Lyar-*KO and WT mESCs. Bright-field images show typical spherical EB morphology in both *Lyar-*KO and WT groups. Fluorescence images reveal stable mCherry expression in *Lyar*-KO EBs, while no fluorescence is detected in WT EBs (n = 3). Scale bar, 250 μm. **(B)** qPCR analysis of lineage-specific differentiation markers in EBs derived from WT and *Lyar*-KO clone 1 and clone 2 at day 0 (D0) and day 6 (D6) of differentiation. Markers for endoderm (*Gata4*, *Sox17*), mesoderm (*Gsc*, *T*), and ectoderm (*Pax6*, *Nestin*) were examined. For each time point, the expression levels of *Lyar*-KO groups were individually normalized to those of the WT group (D0 *Lyar*-KO vs. D0 WT; D6 *Lyar*-KO vs. D6 WT). Data are presented as mean ± SEM (n = 3) and the significance level was calculated by Student’s t test (two-tailed, equal variance) (ns, not statistically significant. *, *p* < 0.05; **, *p* < 0.01; ***, *p* < 0.001; ****, *p* < 0.0001).

To further validate the pluripotency of *Lyar*-KO mESCs, embryoid body (EB) differentiation assays were performed to assess the capacity of mESCs to differentiate into cell types of the three germ layers. During EB formation and differentiation, *Lyar*-KO mESCs maintained stable mCherry expression (Figure 4A), consistent with the persistent integration of the PGK-Puro-P2A-mCherry cassette. To systematically evaluate the trilineage differentiation potential of *Lyar*-KO mESCs, we detected the expression of lineage-specific markers across the three germ layers in EBs from *Lyar*-KO clones 1 and 2, and WT controls at day 0 (D0) and day 6 (D6) using qPCR (Figure 4B). In undifferentiated mESCs at D0, *Lyar*-KO did not alter the expression of NANOG and OCT4 at the protein level (Figures 3A,B), while their transcript levels were significantly reduced. At D0, among all the examined markers, only the ectodermal marker *Pax6* was significantly downregulated in *Lyar*-KO groups relative to WT, while the expression of all other germ layer-specific markers showed no significant difference between KO and WT groups. At D6, complete functional loss of *Lyar* led to marked downregulation of most germ layer-specific markers in both *Lyar*-KO clones relative to WT controls, including all mesodermal markers (*Gsc*, *T*), endodermal markers (*Gata4*, *Sox17*) and the ectodermal markers *Pax6*. For the ectodermal marker *Nestin*, however, a clone-specific expression pattern was observed at D6—its expression showed no significant difference in one *Lyar*-KO clone relative to WT, yet was significantly reduced in the other (∗*p* < 0.05). These findings suggest that *Lyar* deletion impairs the trilineage differentiation potential of mESCs by dysregulating the expression of germ layer-specific markers.

## 4 Discussion

In this study, by generating *Lyar* knockout mESCs, we found that *Lyar* contributes to efficient cell proliferation and proper cell cycle progression. Loss of *Lyar* markedly impairs trilineage differentiation potential by attenuating the expression of lineage-specific markers during EB differentiation. Collectively, these findings identify *Lyar* as a critical regulator acting at the interface between cell cycle progression and early lineage commitment of mESCs.

A key finding of this study is that *Lyar* loss leads to a significant proliferation defect in mESCs, as evidenced by the reduced growth rate in CCK-8 assays (Figure 3C) and elevated G0/G1 phase fraction accompanied by decreased S-phase cells in EdU incorporation assays (Figure 3D). Importantly, these phenotypes were consistently observed in two independent *Lyar*-KO clones, thus excluding clonal variation. Our results are consistent with previous work by Li et al. (2009), who reported impaired proliferation and increased apoptosis in *Lyar*-knockdown mESCs. In line with these findings, our flow cytometric analysis further confirmed significantly elevated apoptosis in *Lyar*-deficient mESCs (Figure 3E), demonstrating that *Lyar* is also required for maintaining mESC survival. To unravel the molecular underpinnings linking *Lyar* deficiency to both cell cycle progression defects and enhanced apoptosis, we focused on key regulatory proteins that govern the G1/S checkpoint and cell survival signaling, including p53, p21, p16, and Cyclin D1. p21 is a direct transcriptional target of p53 and a potent inhibitor of G1 cyclin-dependent kinases (El-Deiry et al., 1993; Harper et al., 1993), which plays a central role in mediating p53-dependent G1 cell cycle arrest. p16 acts as a specific inhibitor of CDK4/6, thereby antagonizing the pro-proliferative activity of cyclin D1, which forms a functional complex with CDK4/6 to drive G1 to S phase transition (Kamb et al., 1994; Quelle et al., 1993; Lukas et al., 1995). Our Western blot results demonstrated that the protein levels of p53 and p21 were significantly elevated, whereas the expression of p16 and cyclin D1 exhibited no obvious changes. These data indicate that the cell cycle progression defect in our model is mainly mediated through the activation of the canonical p53-p21 signaling axis, rather than the p16-cyclin D1 pathway. A checkpoint-deficient, rapid cell cycle is recognized as an intrinsic trait of ESCs which acts in concert with transcription factors to maintain pluripotency (Boheler, 2009). Our findings raise the possibility that *Lyar* serves as an important component of the regulatory network governing the characteristic cell cycle program in mESCs.

Li *et al.* showed that *Lyar* knockdown delays the silencing of pluripotency markers (*Oct4*) and compromises the induction of germ layer markers (*Fgf5*, *Nestin*, *Gata6*, *MHox*) during EB differentiation (Li et al., 2009). Notably, in contrast to the previous *Lyar* knockdown model, our complete *Lyar* knockout model provides genetically more definitive evidence and novel mechanistic insights into *Lyar*’s function in mESCs. Most importantly, we identified and validated the molecular mechanism underlying cell cycle regulation by *Lyar*, which was not observed in the knockdown model. In addition, this study also provides a more comprehensive analysis of *Lyar*’s function in regulating trilineage differentiation. Extending these findings, our qPCR data at day 6 of EB differentiation reveal that the complete functional loss of *Lyar* results in significant downregulation of most lineage-specific markers across the three germ layers in two independent *Lyar*-KO clones: mesoderm (*Gsc*, *T*) and endoderm (*Gata4*, *Sox17*) markers show a general downregulation, and the ectodermal markers *Pax6* is notably decreased, with only *Nestin* exhibiting clone-specific expression variation in the KO clones (Figure 4B). This discrepancy from Li et al. (2009)—who reported reduced *Nestin* expression—likely arises from the distinct experimental models employed (knockdown versus knockout) or the specific differentiation time points analyzed. As *Nestin* is an early neural stem cell marker (Lendahl et al., 1990), whereas *Pax6* marks committed neural progenitors and is expressed subsequent to early neural markers such as *Sox1* and *Nestin* during differentiation (Bylund et al., 2003; Mori et al., 2005; Suter et al., 2009), these observations suggest that *Lyar* preferentially regulates the specification of *Pax6*-positive neural lineages rather than the initial induction of *Nestin*-positive cells. Additionally, the impaired proliferation in *Lyar*-KO mESCs may indirectly contribute to this differentiation defect. Indeed, the downregulated expression of lineage-specific markers during EB differentiation is mainly caused by impaired G1 phase progression rather than a direct regulatory effect on differentiation programs, since rapid progression through the G1 phase is tightly coupled to the maintenance of pluripotency and the initiation of lineage commitment in ESCs (Becker et al., 2010; Becker et al., 2006).

Nevertheless, a limitation of the present study should be acknowledged. Our conclusion that *Lyar* deficiency impairs the trilineage differentiation potential of mESCs is based solely on qPCR analysis of a limited number of germ layer-specific markers at a single time point during EB differentiation. Further functional differentiation assays and protein-level validation would be required to provide more robust and definitive evidence in future studies.

In conclusion, this study confirms and extends previous findings by demonstrating that *Lyar* is a critical regulator of mESC proliferation, survival, and differentiation. Specifically, *Lyar* contributes to proper cell cycle progression to support proliferation, and loss of *Lyar* leads to elevated apoptosis via activation of the p53-p21 pathway. Moreover, *Lyar* deletion impairs trilineage differentiation potential by dysregulating germ layer-specific marker expression. These findings enhance our understanding of the complex regulatory network governing ESC biology and may have implications for optimizing ESC-based regenerative medicine strategies.

## Data Availability

The original contributions presented in the study are included in the article/Supplementary Material, further inquiries can be directed to the corresponding author.
