# Supplementary material for: Lyar contributes to cell cycle progression and multi-lineage differentiation in mouse embryonic stem cells
Source: Front Genet. 2026 Mar 20;17:1786528. doi: 10.3389/fgene.2026.1786528 (PMC13047206; doi:10.3389/fgene.2026.1786528)
Supplement: Supplementary file 1 [file DataSheet1.pdf]

Supplementary material

***Lyar* contributes to cell cycle progression and multi-lineage differentiation in mouse embryonic stem cells**

Yuanqing Pan, Yuqi Su, Li Xing, Mingze Yao

Uncropped Western blot

Fig.1C

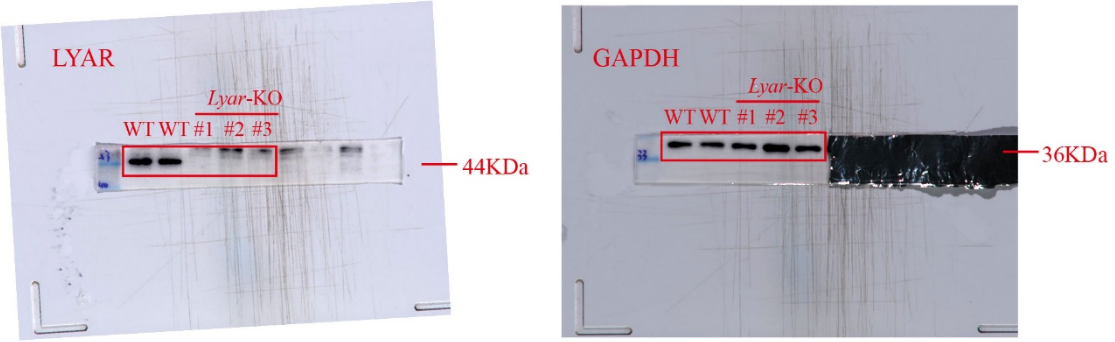

Marker: Sangon Biotech TureColor Pre-stained Protein Marker, 3 colors (10-180kDa) NO.C510010

Fig.3F

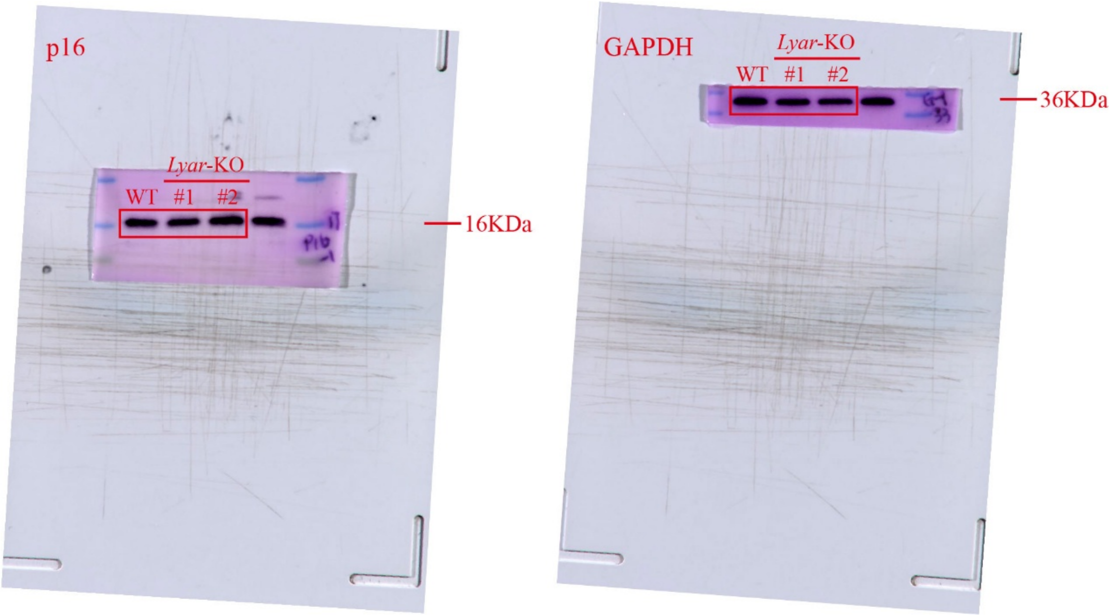

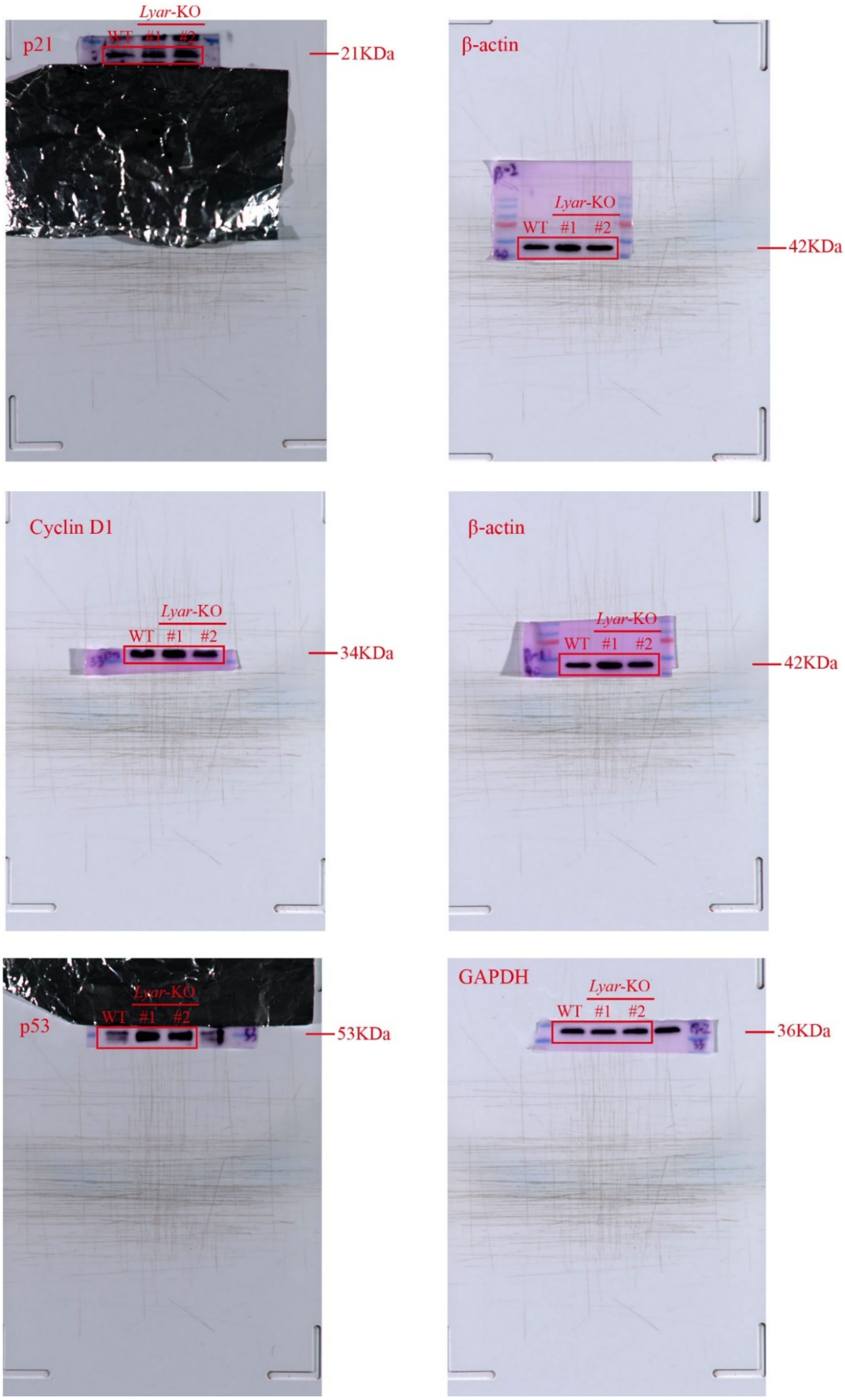

Marker: Sangon Biotech TureColor Pre-stained Protein Marker, 3 colors (10-180kDa)
NO.C510010
